# Alum Adjuvant and Built-in TLR7 Agonist Synergistically Enhance Anti-MUC1 Immune Responses for Cancer Vaccine

**DOI:** 10.3389/fimmu.2022.857779

**Published:** 2022-03-16

**Authors:** Shi-Hao Zhou, Yu-Ting Li, Ru-Yan Zhang, Yan-Ling Liu, Zi-Wei You, Miao-Miao Bian, Yu Wen, Jian Wang, Jing-Jing Du, Jun Guo

**Affiliations:** ^1^ Key Laboratory of Pesticide and Chemical Biology of Ministry of Education, Hubei International Scientific and Technological Cooperation Base of Pesticide and Green Synthesis, International Joint Research Center for Intelligent Bio-sensing Technology and Health, College of Chemistry, Central China Normal University, Wuhan, China; ^2^ Hubei Key Laboratory of Kidney Disease Pathogenesis and Intervention, School of Medicine, Hubei Polytechnic University, Huangshi, China

**Keywords:** MUC1 glycopeptide, TLR7 agonist, alum adjuvant, synergistic effect, cancer vaccine

## Abstract

The tumor-associated antigen mucin 1 (MUC1) is an attractive target of antitumor vaccine, but its weak immunogenicity is a big challenge for the development of vaccine. In order to enhance immune responses against MUC1, herein, we conjugated small molecular toll-like receptor 7 agonist (TLR7a) to carrier protein BSA *via* MUC1 glycopeptide to form a three-component conjugate (BSA-MUC1-TLR7a). Furthermore, we combined the three-component conjugate with Alum adjuvant to explore their synergistic effects. The immunological studies indicated that Alum adjuvant and built-in TLR7a synergistically enhanced anti-MUC1 antibody responses and showed Th1-biased immune responses. Meanwhile, antibodies elicited by the vaccine candidate effectively recognized tumor cells and induced complement-dependent cytotoxicity. In addition, Alum adjuvant and built-in TLR7a synergistically enhanced MUC1 glycopeptide-specific memory CD8+ T-cell immune responses. More importantly, the vaccine with the binary adjuvant can significantly inhibit tumor growth and prolong the survival time of mice in the tumor challenge experiment. This novel vaccine construct provides an effective strategy to develop antitumor vaccines.

## Introduction

Tumor-associated antigen mucin 1 (MUC1) is overexpressed on many human epithelial tumor tissues, such as breast, ovarian, and prostate carcinomas, which makes it an attractive target for tumor immunotherapy (1, 2). MUC1 contains a variable number of tandem repeat (VNTR) sequence (HGVTSAPDTRPAPGSTAPPA), and five potential O-glycosylation sites are located on the threonines and serines of the tandem repeat sequence (3–5). In normal cells, MUC1 glycoproteins are expressed in a polarized fashion, whereas MUC1 glycoproteins in tumor cells are overexpressed with much less glycosylation, present all over the cell surface, and show an unpolarized fashion (6, 7). MUC1 glycopeptide from VNTR sequence was often used as the antigen for the MUC1-targeted antitumor vaccines (8–13). However, due to the weak immunogenicity of MUC1 glycopeptide, appropriate immune stimulators or carriers are necessary in vaccine designs (4, 14).

Toll-like receptors (TLRs) are pattern-recognition receptors (PRRs) found in many immune cells such as macrophages and dendritic cells (DCs) (15–18). TLRs can recognize nucleic acid molecules or cell wall components of pathogens to the innate immune system, and then regulate the activation of antigen-presenting cells (APCs) (19–21). The purine-rich RNA is a natural ligand for TLR7, which induces the innate immune response to the invading pathogen (22–25). Due to the important role of TLR7 in activating the immune system, many studies focused on the conjugation of TLR7 agonists to antigens for enhancing immune responses (26–29).

The most widely used Alum adjuvant in human vaccines has the great potential to adsorb antigens and alter their pharmacokinetic and immunological properties (30). Many studies used Alum adjuvant as immunostimulatory in vaccine designs to enhance the immunogenicity of antigens (31–33). However, Alum adjuvant alone mainly stimulates the Th2-biased immune responses and induces relatively weak cellular immune responses (30, 34, 35). To deal with this challenge, some researchers used Alum adjuvant combined physically with other adjuvants that could induce Th-1 immune responses (36–39).

In the design of semisynthetic vaccines, MUC1 glycopeptide was often conjugated to the carrier proteins such as bovine serum albumin (BSA), keyhole limpet hemocyanin (KLH), and tetanus toxoid (TT) (40–42). On the one hand, semisynthetic vaccines containing carrier protein are usually facile to prepare and could effectively induce both humoral and cellular immune responses (43–45). On the other hand, the carrier proteins provide multiple Th and Tc epitopes that could synergistically activate the adaptive immune response (14, 46).

**Scheme 1:**
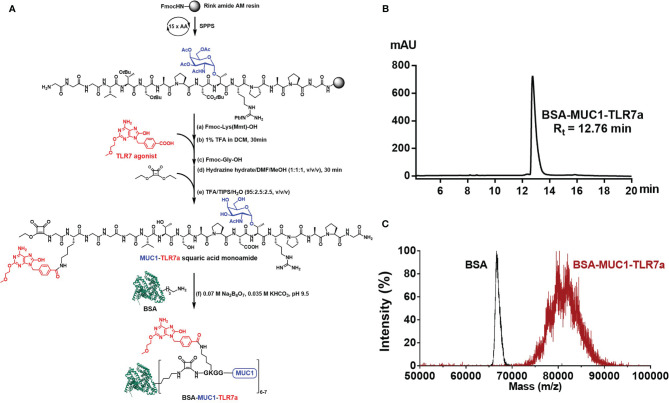
**(A)** Synthesis of BSA-MUC1-TLR7a conjugate. **(B)** The HPLC analysis result showed that the purity of BSA-MUC1-TLR7a conjugate was higher than 95%. **(C)** The MALDI-TOF-MS result showed that each BSA protein conjugated an average of 6-7 MUC1-TLR7a glycopeptides.

**Figure 1:**
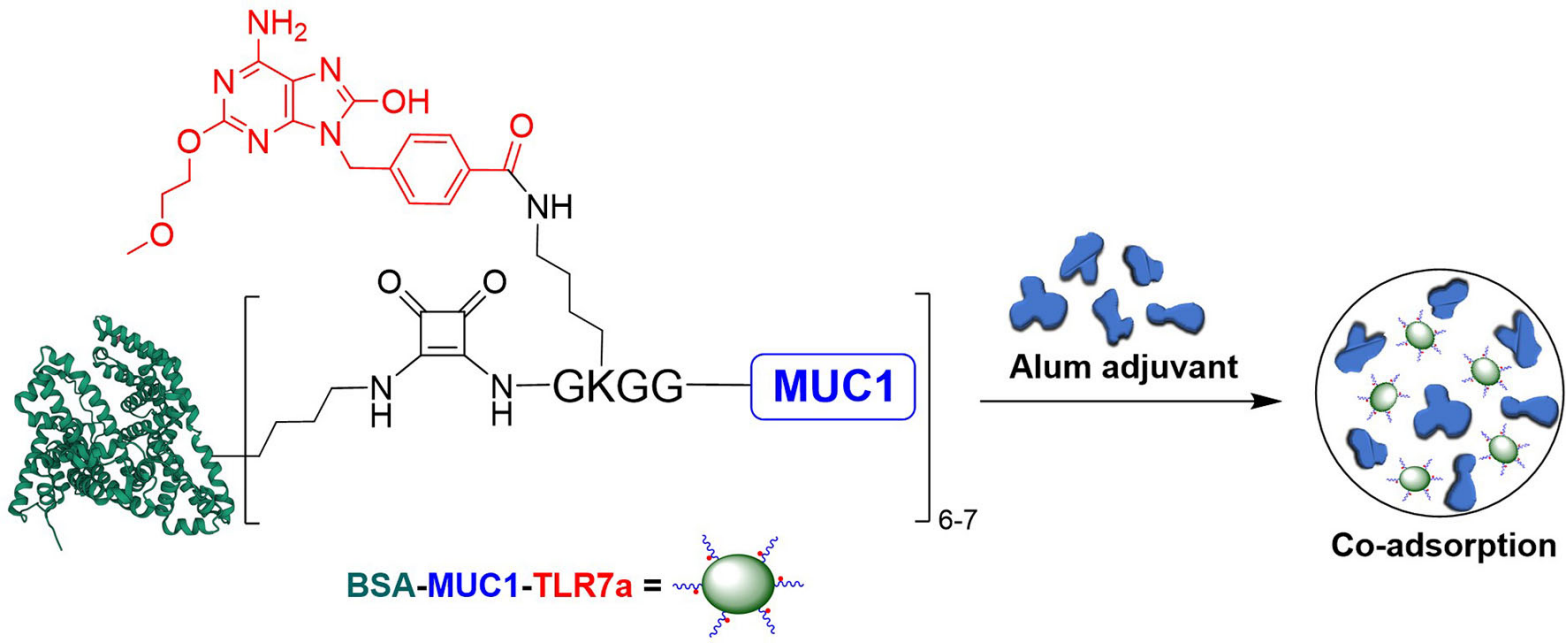
Design of the three-component conjugate (BSA-MUC1-TLR7a) and its co-adsorption with Alum adjuvant (PDB code of BSA: 4F5S).

Based on these considerations, we covalently conjugated TLR7 agonist (TLR7a) to MUC1 glycopeptide through solid-phase synthesis method (SPPS) in our vaccine design (**Scheme 1A**). The glycopeptide-adjuvant conjugate MUC1-TLR7a was then covalently linked to the carrier protein BSA through the squaric acid diethyl ester method to form a three-component conjugate (BSA-MUC1-TLR7a). In this strategy, MUC1 and TLR7a can be easily covalently conjugated to the carrier protein through only one step, and the molar ratio of MUC1 and TLR7a was strictly controlled at 1:1, which could avoid the uncertainty caused by the random coupling of adjuvant and antigen. In addition, the strategy of built-in TLR7 agonist could also avoid uncontrolled systemic toxicity caused by the diffusion of small molecule (22, 47). Furthermore, the BSA-MUC1-TLR7a conjugate was combined with Alum adjuvant to determine whether they could synergistically enhance the anti-MUC1-specific immune responses (**Figure 1**). This is the first time to introduce Alum adjuvant to the self-adjuvanting protein conjugate in the MUC1-targeted antitumor vaccine.

## Results and Discussion

### Synthesis of BSA-MUC1-TLR7a Conjugate

Here, we designed a new vaccine construction based on TLR7 agonist for tumor immunotherapy. In this strategy, MUC1 glycopeptide (GVTSAPDTRPAPG, 13aa) from the VNTR of MUC1 glycoprotein was used as an antigen, which was synthesized through the solid-phase peptide synthesis (SPPS) with Rink Amide-AM Resin (**Scheme 1A**). With glycine as spacer, the Fmoc-Lys(Mmt)-OH was conjugated to the resin-bound peptide. After deprotection of Mmt protection group with 1% trifluoroacetic acid (TFA) in DCM, the TLR7a was coupled to the amino group of the lysine side chain. Then, after the coupling of glycine and diethyl squarate, the TLR7a-MUC1 glycopeptide squaric acid monoamide was synthesized after removal from the resin and deprotection. The product was purified by high-performance liquid chromatography (HPLC, **Figure S4**) and analyzed by high-resolution mass spectrometry (HRMS, **Figure S5**). Finally, the TLR7a-MUC1 glycopeptide squaric acid monoamide and BSA carrier protein were dissolved and mixed in Na_2_B_4_O_7_/KHCO_3_ buffer (pH = 9.5) to form BSA-MUC1-TLR7a conjugate. The conjugate was purified by size-exclusion gel filtration (30 kD) and analyzed by HPLC (**Scheme 1B**). The average capacity of TLR7a-MUC1 on BSA carrier protein (average of 6–7) was estimated by matrix-assisted laser desorption/ionization time-of-flight mass (MALDI-TOF-MS, **Scheme 1C**).

### Vaccine Design and Immunization

Six MUC1-targeted antitumor vaccine candidates were designed: (A) BSA-MUC1 alone; (B) BSA-MUC1 mixed with TLR7a; (C) BSA-MUC1 mixed with Alum adjuvant; (D) BSA-MUC1 mixed with TLR7a and Alum adjuvant; (E) BSA-MUC1-TLR7a; and (F) BSA-MUC1-TLR7a mixed with Alum adjuvant. All vaccine candidates contain 10 nmol MUC1glycopeptide or 10 nmol TLR7 agonist. In these vaccine candidates, two important factors were considered: the covalent coupling of TLR7 agonist and the use of Alum adjuvant (**Table S1**).

Female BALB/c mice aged 6 weeks were vaccinated on days 1, 15, and 29 for subcutaneous injection (200 μL per mouse), and the sera were collected on days 14, 28, and 42 to evaluate the antibody immune responses including enzyme linked immunosorbent assay (ELISA), fluorescence-activated cell sorting (FACS) analysis, and complement-dependent cytotoxicity (CDC) assay (48–50). In addition, the spleens from mice were collected on days 42 to evaluate the cellular immune responses including cytotoxic T lymphocyte (CTL) assay, intracellular cytokine staining (ICS) assay, and enzyme-linked immunospot (ELISpot) assay (51–54). All mice used in the experiments were purchased and bred in the Laboratory Animal Centre of Huazhong Agriculture University, and the experiments were conducted strictly in accordance with the principles of welfare and ethics of medical laboratory animals.

### Evaluation of Cytokine Levels and Antigen-Specific Antibodies

**Figure 2:**
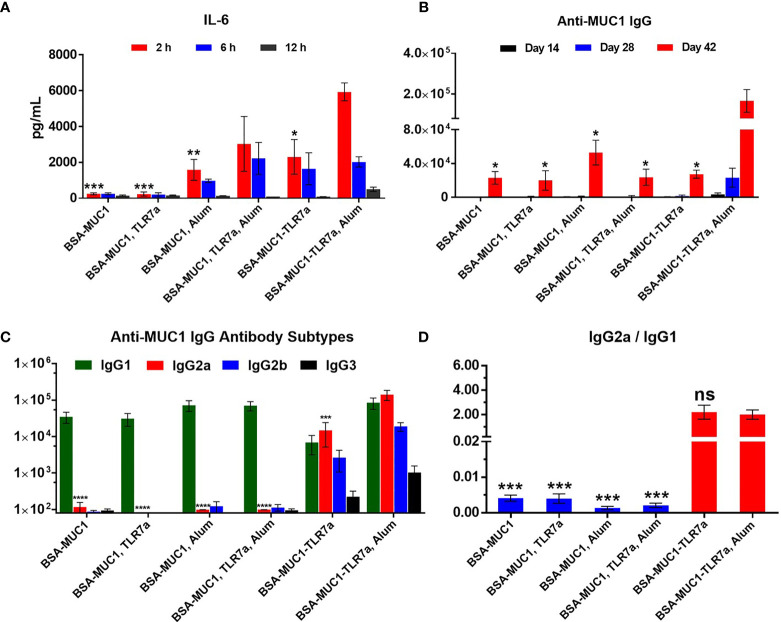
**(A)** The secretion of the IL-6 cytokines in serum samples from immunized BALB/c mice with vaccine candidates at 2, 6, and 12 h, respectively. **(B)** Anti-MUC1 IgG antibody titers of each group on days 14, 28, and 42. **(C)** MUC1-specific IgG antibody subtypes on day 42 and **(D)** the ratio of IgG2a/IgG1 of mice. The data are expressed as the mean ± SEM. Asterisks show significant difference compared with BSA-MUC1-TLR7a/Alum group based on one-way ANOVA by Dunn’s multiple comparison test (no significant difference, ns; ^*^ *p* ≤ 0.05, ^**^ *p* ≤ 0.01; ^***^ *p* ≤ 0.001; ^****^ *p* ≤ 0.0001).

IL-6 cytokine is the representative of inflammatory cytokines; the secretion of IL-6 cytokines in the sera of mice was measured to determine whether the vaccine candidates could effectively activate the immune system (27). Mice were injected with vaccine candidates and sera were collected at 2, 6, and 12 h after immunization. As shown in **Figure 2A**, both the built-in TLR7 agonist and Alum adjuvant induced the secretion of IL-6 cytokines (2,303 and 1,586 pg/ml at 2 h, respectively). In particular, when BSA-MUC1-TLR7a was combined with Alum adjuvant, higher levels of IL-6 cytokines were detected in the sera of mice (5,932 pg/ml at 2 h). Although there is no significant difference, the secretion levels of IL-6 cytokines of BSA-MUC1/TLR7a/Alum group (3,034 pg/ml at 2 h) were still lower compared with BSA-MUC1-TLR7a/Alum group. These results indicated that the combination of built-in TLR7 agonist and Alum adjuvant could synergistically stimulate the immune system and induce higher levels of IL-6 cytokines. Interestingly, higher levels of IL-6 cytokines were still detected in the sera of BSA-MUC1-TLR7a/Alum group at 12 h after vaccination. This result could be attributed to the adsorption of proteins by Alum adjuvant, showing a slow release and continuous stimulation to the immune system.

The level of induced specific antibodies is an important index to evaluate the efficacy of vaccine candidates. As shown in **Figure 2B**, the built-in TLR7 agonist and Alum adjuvant could synergistically induce higher anti-MUC1 IgG antibody titer (166,809) on day 42 compared with other vaccine candidates. In addition, the experimental result showed that physical mixing of TLR7 agonist with BSA-MUC1 slightly decreased the anti-MUC1 IgG antibody titer (23,078 and 20,063 for the BSA-MUC1 alone and mixed with TLR7a, respectively) on day 42, which may be due to the uncontrolled systemic toxicity caused by the diffusion of small molecules TLR7 agonist. Meanwhile, compared with BSA-MUC1, BSA-MUC1/Alum only induced about 2-fold higher anti-MUC1 IgG antibody titer (52,943) on day 42. Built-in TLR7 slightly increased IgG antibody titer (27,431), which is somewhat different from previous work and may be due to the dfferences in coupling strategy (46). Thus it can be seen that the synergistic effect of built-in TLR7 agonist and Alum adjuvant is the key factor to enhance antibody immune responses in this strategy. This may be attributed to the adsorption of Alum adjuvant to BSA-MUC1-TLR7a conjugate, which plays a slow release effect. Meanwhile, the built-in TLR7 agonist could also avoid uncontrolled systemic toxicity caused by the diffusion of small molecules (see **Figures S6, S7** for IgM and anti-BSA antibody titers).

In terms of IgG antibody subtypes, as shown in **Figure 2C**, BSA-MUC1 alone and BSA-MUC1 mixed with Alum adjuvant or TLR7 agonist mainly induced IgG1 antibody and hardly caused the production of IgG2a, IgG2b, and IgG3 antibodies, showing a strong Th2-biased immune responses with low ratio of IgG2a/IgG1 (**Figure 2D**). Although the built-in TLR7a only slightly increase the anti-MUC1 IgG antibody titer, but it significantly improved the ratio of IgG2a/IgG1 (2.2) and showed a Th1-biased immune responses. Whereas the BSA-MUC1-TLR7a combined with Alum adjuvant not only induced IgG1 antibody, but it also elicited high levels of IgG2a and IgG2b antibodies, showing a Th1-biased immune responses (IgG2a/IgG1 = 2).

### Immunological Studies With Cancer Cells

**Figure 3:**
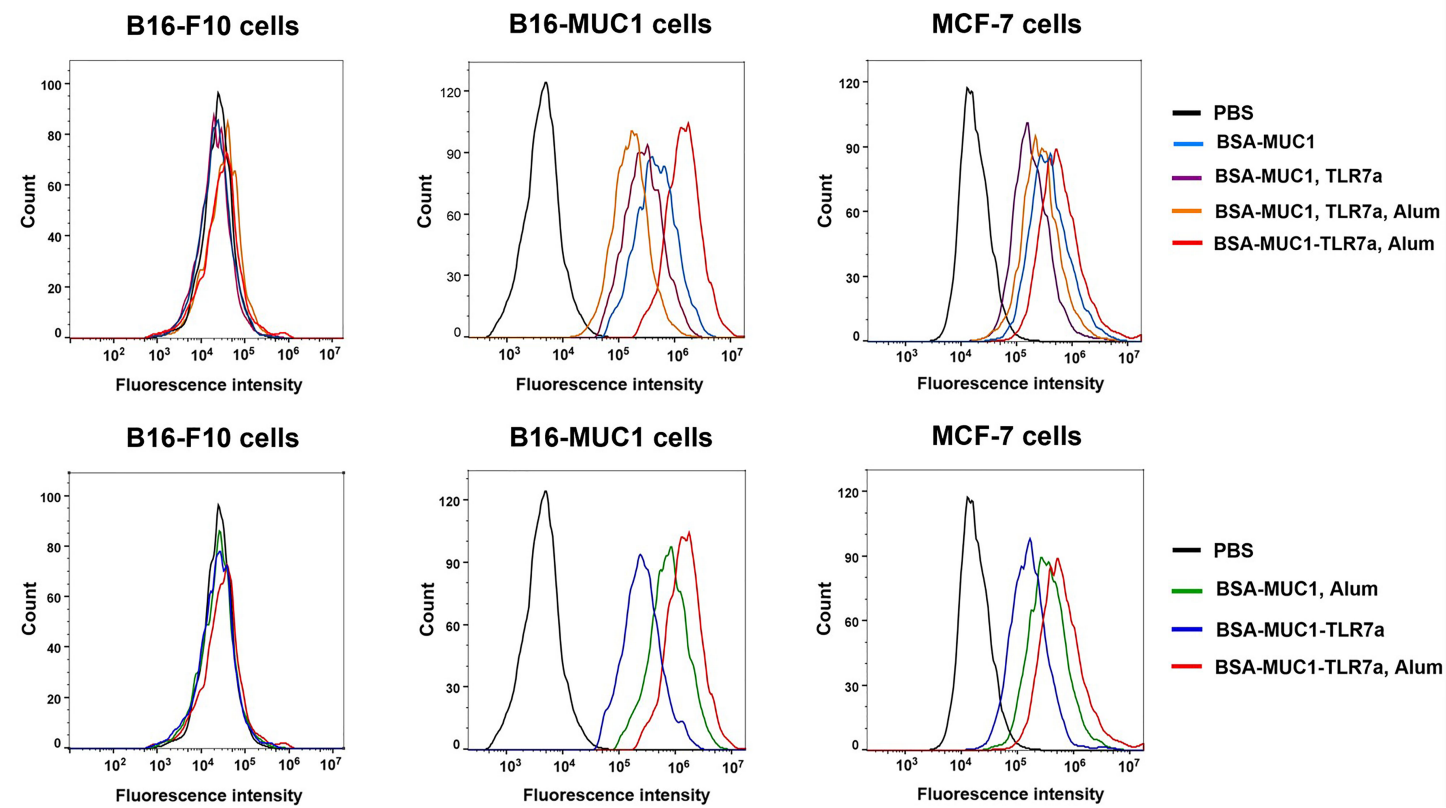
Specific antibodies recognized and bound MUC1-positive MCF-7 and B16-MUC1 cells instead of B16-F10 cells.

To determine whether induced antibodies can effectively recognize and bind to target tumor cells, MUC1-positive MCF-7 and B16-F10 cells were incubated with pooled sera of each group and B16-F10 cells were used as negative control cells (46). After washing, cells were incubated with Alexa Fluor 488-conjugated goat anti-mouse IgG and analyzed by flow cytometry. As shown in **Figure 3**, the results were consistent with the previous trend of anti-MUC1 IgG antibody titers, the serum antibodies from mice immunized with BSA-MUC1-TLR7a/Alum could effectively recognize and bind MUC1-positive tumor cells including MCF-7 and B16-MUC1 cells. Furthermore, serum antibodies from all groups could not significantly bind B16-F10 cells, indicating that the binding of antibodies to MCF-7 and B16-MUC1 cells was MUC1-targeted.

**Figure 4:**
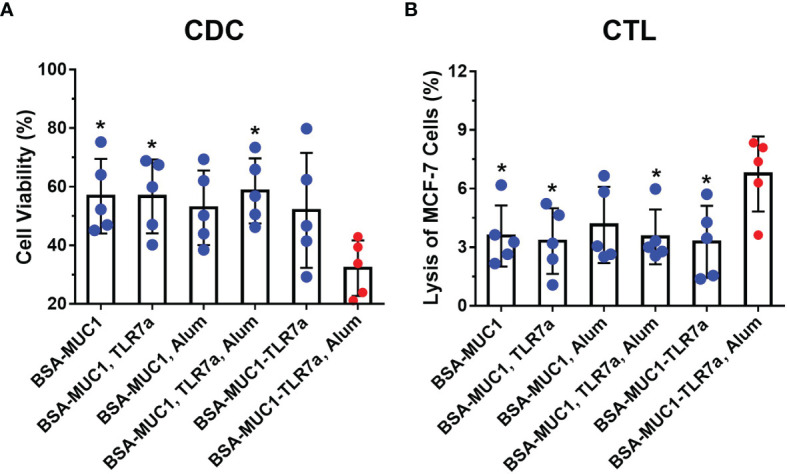
**(A)** The complement-dependent cytotoxicity (CDC) assay of sera from vaccinated mice (MCF-7 cells). **(B)** The cytotoxic T-lymphocyte (CTL) assay of spleen from vaccinated mice on day 42. The data are expressed as the mean ± SEM. Asterisks show significant difference compared with BSA-MUC1-TLR7a/Alum group based on one-way ANOVA by Dunn’s multiple comparison test (*P ≤ 0.05).

To assess the ability of antibodies to activate the complement system, the complement-dependent cytotoxicity (CDC) assay was performed by the tetrazolium bromide (MTT) test (48, 55). As shown in **Figure 4A**, the serum antibodies from BSA-MUC1-TLR7a/Alum group cannot only effectively bind MCF-7 cells but also effectively cause complement-dependent cytotoxicity to kill target cells (cell viability was 56% for BSA-MUC1 alone, 56% for mixing with TLR7a, 53% for mixing Alum adjuvant, 58% for mixing with TLR7a and Alum adjuvant, 52% for BSA-MUC1-TLR7a, and 32% for the BSA-MUC1-TLR7a mixed with Alum adjuvant, respectively). Furthermore, to investigate whether the candidate vaccines elicit cytotoxic T lymphocyte (CTL) response to kill target cells, splenocytes were obtained from immunized mice on day 42 and incubated with MCF-7 cancer cells (46, 56). The lysis of MCF-7 cells was then determined by lactate dehydrogenase (LDH) assay. As shown in **Figure 4B**, the results showed that the cytotoxicity of splenocytes of BSA-MUC1-TLR7a/Alum group was significantly higher than that of other groups (lysis of MCF-7 cells was 3.6% for BSA-MUC1 alone, 3.3% for mixing with TLR7a, 4.1% for mixing Alum adjuvant, 3.5% for mixing with TLR7a and Alum adjuvant, 3.3% for BSA-MUC1-TLR7a, and 6.8% for the BSA-MUC1-TLR7a mixed with Alum adjuvant, respectively).

### Cellular Immune Responses

**Figure 5:**
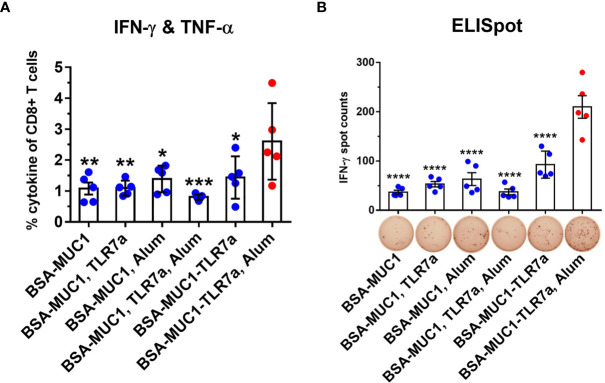
Lymphocytes in the spleen (on day 42) were collected and stimulated with the MUC1 glycopeptide. Then the ICS assay **(A)** and ELISpot assay **(B)** were performed. The data are expressed as the mean ± SEM. Asterisks show significant difference compared with BSA-MUC1-TLR7a/Alum group based on one-way ANOVA by Dunn’s multiple comparison test (^*^ *p* ≤ 0.05; ^**^ *p* ≤ 0.01; ^***^ *p* ≤ 0.001; ^****^ *p* ≤ 0.0001).

Cellular immune response is another important indicator to evaluate cancer vaccines, which may also provide insight into mechanisms beyond MUC1-specific immune responses that are promoting antitumor activity *in vivo.* In order to evaluate cellular immune responses after vaccination, lymphocytes in the spleen were stimulated with the MUC1 glycopeptide, then the results were analyzed by intracellular cytokine staining (ICS) assay including tumor necrosis factor-α (TNF-α) and gamma interferon (IFN-γ). MUC1-reactive memory of CD8+ T cells was analyzed by flow cytometry (**Figures 5A** and **S8**). Compared with other groups, immunization with BSA-MUC1-TLR7a/Alum induced more CD8+ T cells that produced Th1 cytokines IFN-γ and TNF-α (1.0% for BSA-MUC1 alone, 1.1% for mixing with TLR7a, 1.4% for mixing Alum adjuvant, 0.8% for mixing with TLR7a and Alum adjuvant, 1.4% for BSA-MUC1-TLR7a, and 2.6% for the BSA-MUC1-TLR7a mixed with Alum adjuvant, respectively). In addition, IFN-γ was also assessed by enzyme-linked immunospot (ELISpot) assay (**Figure 5B**). The results showed that vaccination with BSA-MUC1-TLR7a/Alum developed higher number of IFN-γ spots than other groups (37 for BSA-MUC1 alone, 53 for mixing with TLR7a, 63 for mixing Alum adjuvant, 37 for mixing with TLR7a and Alum adjuvant, 93 for BSA-MUC1-TLR7a, and 210 for the BSA-MUC1-TLR7a mixed with Alum adjuvant, respectively). All the experimental results showed that built-in TLR7a agonist and Alum adjuvant could synergistically induce a stronger immune response than other groups.

### Evaluation of Antitumor Immune Response in Mice

**Figure 6:**
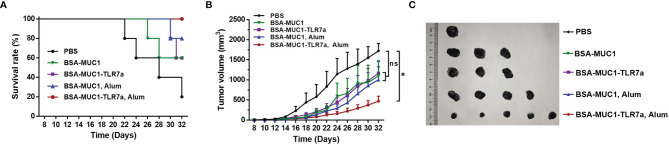
Survival rate of mice **(A)** and tumor growth **(B)** of mice injected with different samples. **(C)** Tumor images of mice euthanized. Asterisks show significant difference based on two-way ANOVA by Dunn’s multiple comparison test compared with PBS group (no significant difference, ns; *P ≤ 0.05).

Furthermore, to determine whether the BSA-MUC1-TLR7a combined with Alum adjuvant can effectively inhibit tumor growth, female C57BL/6 mice aged 6 weeks were challenged by subcutaneous injection of 5 × 10^5^ B16-MUC1 cells per mouse. The mice were inoculated with the vaccines on days 12, 16, and 20 for three times in total (5 mice per group). As shown in **Figures 6B, C**, the mean tumor volume of BSA-MUC1-TLR7a/Alum group was less than 500 mm^3^ on day 32, while the mean tumor volume of the other groups exceeded 1,000 mm^3^. Meanwhile, the analysis results showed that only the BSA-MUC1-TLR7/Alum group could significantly inhibit the tumor growth compared with PBS group, and there was no significant difference between PBS group and other groups. The results showed that the BSA-MUC1-TLR7a combined with Alum adjuvant can effectively inhibit tumor growth and prolong the survival time of mice (**Figure 6A**), indicating that this conjugate vaccine containing Alum adjuvant is a promising vaccine candidate inducing effective anti-tumor immune responses in mice.

## Conclusion

Herein, we report for the first time that the self-adjuvanting protein conjugate is combined with Alum adjuvant in MUC1-targeted antitumor vaccine, which induced potent immune responses. In this strategy, the TLR7 agonist was conjugated to carrier protein *via* MUC1 glycopeptide, which could avoid systemic toxicity caused by the diffusion of small molecules. On the one hand, the experimental results indicated that BSA-MUC1-TLR7a/Alum induced high anti-MUC1 IgG antibody titers and showed a Th1-biased immune response. Furthermore, the serum antibody induced by the BSA-MUC1-TLR7a/Alum can effectively bind MCF-7 cells and cause complement dependent cytotoxicity. On the other hand, ICS and ELISpot assays also determined that BSA-MUC1-TLR7a/Alum induced stronger cellular immune responses compared with other groups. More importantly, BSA-MUC1-TLR7a/Alum can significantly inhibit tumor growth and prolong the survival time of the tumor-bearing mice. Thus, this vaccine design represents an effective strategy for tumor immunotherapy and may provide a potential strategy against other diseases.

## Data Availability Statement

The original contributions presented in the study are included in the article/**Supplementary Material**. Further inquiries can be directed to the corresponding author.

## Ethics Statement

The animal study was reviewed and approved by the Ethics Review Committee for Life Science Study of Central China Normal University.

## Author Contributions

JG and S-HZ conceived the research ideas, supervised the project, and wrote the manuscript. Y-TL, Y-LL, Z-WY, and M-MB performed the animal experiments. R-YZ, JW, J-JD, and YW performed the immunological experiments. All authors discussed the results and commented on the manuscript. All authors listed have made a substantial, direct, and intellectual contribution to the work and approved it for publication.

## Funding

The project was funded by the National Natural Science Foundation of China (22177035, 21772056), the National Key Research and Development Program of China (2017YFA0505200), Wuhan Bureau of Science and Technology (2020020601012217), the self-determined research funds of CCNU from the colleges’ basic research and operation of MOE (CCNU20TS016), and Program of Introducing Talents of Discipline to Universities of China (111 program, B17019).

## Conflict of Interest

The authors declare that the research was conducted in the absence of any commercial or financial relationships that could be construed as a potential conflict of interest.

## Publisher’s Note

All claims expressed in this article are solely those of the authors and do not necessarily represent those of their affiliated organizations, or those of the publisher, the editors and the reviewers. Any product that may be evaluated in this article, or claim that may be made by its manufacturer, is not guaranteed or endorsed by the publisher.
