# Supplementary material for: Alum Adjuvant and Built-in TLR7 Agonist Synergistically Enhance Anti-MUC1 Immune Responses for Cancer Vaccine
Source: Front Immunol. 2022 Mar 16;13:857779. doi: 10.3389/fimmu.2022.857779 (PMC8965739; doi:10.3389/fimmu.2022.857779)
Supplement: Supplementary file 1 [file DataSheet_1.pdf]

Supplementary Material

Alum Adjuvant and Built-in TLR7 Agonist Synergistically Enhance Anti-MUC1 Immune Responses for Cancer Vaccine

Shi-Hao Zhou, Yu-Ting Li, Ru-Yan Zhang, Yan-Ling Liu, Zi-Wei You, Miao-Miao Bian, Yu Wen, Jian Wang, Jing-Jing Du, and Jun Guo[*]

This file includes:

General information and synthetic methods

Evaluation of vaccine candidates

Biological testing methods

Supplemental Schemes S1 to S2

Supplemental Table S1

Supplemental Figures S1 to S10

Contents:

1. **General information and synthetic methods** S3

   1.1 General information S3

   1.2 Synthesis of the BSA-MUC1 .... S4

   1.3 Synthesis of the BSA-MUC1-TLR7a S6

2. **Evaluation of vaccine candidates** S7

   2.1 Vaccination formulation S7

   2.2 Immunological evaluation S7

3. **Biological testing methods** S9

   3.1 Immunization of mice and ELISA S9

   3.2 Cell culture and FACS assay S10

   3.3 Complement dependent cytotoxicity (CDC) assay S11

   3.4 Cytotoxic T lymphocyte assay (CTL) assay S11

   3.5 Enzyme-linked immunospot (ELISpot) assay S11

   3.6 Intracellular cytokine staining (ICS) S12

   3.7 Evaluation of anti-tumor immune response in mice S12

   3.8 Statistical analysis and references S12

1. General information and synthetic methods

1.1 General information

All Fmoc L-amino acids and pre-loaded resins were purchased from GL Biochem. Anhydrous dichloromethane (DCM) was obtained from the drying solvent system (passed through CaH_2) and can be used without further drying. The purchased anhydrous dimethylformamide (DMF) was stored over 4Å molecular sieves. Methanol, piperidine and hydrazine hydrate were purchased from Sinopharm Chemical Reagent (Shanghai, China). Benzotriazol-1-yl-oxytripyrrolidinophosphonium hexafluorophosphate (PyBOP), trifluoroacetic acid (TFA), *O*-(7-azabenzotriazol-1-yl)-*N*,*N*,*N'*,*N'*-tetramethyluronium hexafluorophosphate (HATU) and 1-hydroxy-7-azabenzotriazole (HOAt) were purchased from Bidepharm (Shanghai, China). *N*,*N*-Diisopropylethylamine (DIPEA), triisopropylsilane (TIPS) and diethyl squarate were purchased from Energy Chemical (Shanghai, China). TLR7 agonist and Biotin-MUC1 glycopeptide were synthesized by the method reported previously (Du et al., 2020). Bovine serum albumin (BSA) and inject^{TM} Alum adjuvant was purchased from Thermo Scientific. Affinipure goat anti-mouse kappa antibody IgG, IgM were purchased from Jackson Immuno Research. Peroxidase-conjugated Affinipure goat anti-mouse kappa IgG1, IgG2a, IgG2b and IgG3 antibodies were purchased from Southern Biotechnology. Semi-preparative HPLC separations were performed on an Agilent 1260 infinity II prime LC system equipped with a C18 column (Agilent, 250 × 9.2 mm, 5 μm) with a binary mixture of solvent A (100% water with 0.1% trifluoroacetic acid) and solvent B (100% acetonitrile HPLC-grade with 0.1% trifluoroacetic acid) as the mobile phase (flow rates of 4.0 mL/min). The HRMS was perfomed on Bruker Compact TOF mass spectrometer by ESI. Matrix-assisted laser desorption/ionization time of flight (MALDI-TOF) MS was performed on an AB SCIEX 5800 spectrometer (Shimadzu AXIMA Assurance). Female BALB/c mice (age 6-8 weeks) were purchased and bred in the Laboratory Animal Centre of Huazhong Agriculture University. All animal experiments were performed in accordance with the principles of welfare and ethics of medical laboratory animals.

1.2 Synthesis of the BSA-MUC1 conjugate

MUC1 glycopeptides were synthesized according to the Fmoc-strategy SPPS starting from Rink amide AM resin. The amino acids (3 Eq.) were coupled using PyBOP (3 Eq.) and DIPEA (6 Eq.) in DMF for 1 h. The Fmoc-glycosyl amino acid (Tn antigen) (1.5 Eq.) was coupled using HATU (1.5 Eq.), HOAt (1.5 Eq.) and DIPEA (3 Eq.) in DMF for 4 h, residual free amines were acetylated with capping reagents. After completion of the glycopeptide assembly, the *N*-terminal Fmoc protecting groups or acetyl moieties of glycosyl amino acids were removed using 20% piperidine in DMF or approximately 30% hydrazine in DMF and MeOH. The linker of MUC1-glycopeptide with a squaric acid diethyl ester at the *N*-terminus was achieved by treatment with DIPEA (5 Eq.) in DMF for 2 h. The resin was treated with the cleavage cocktail (trifluoroacetic acid 95%, triisopropylsilane 2.5%, water 2.5%, 10 mL) for 2 h. The resin was filtered, washed three times with trifluoroacetic acid, the residue was concentrated and precipitated with diethyl ether, the crude MUC1 glycopeptides were purified by semi-preparative HPLC (Figure S1) and identified with HRMS (Figure S2).

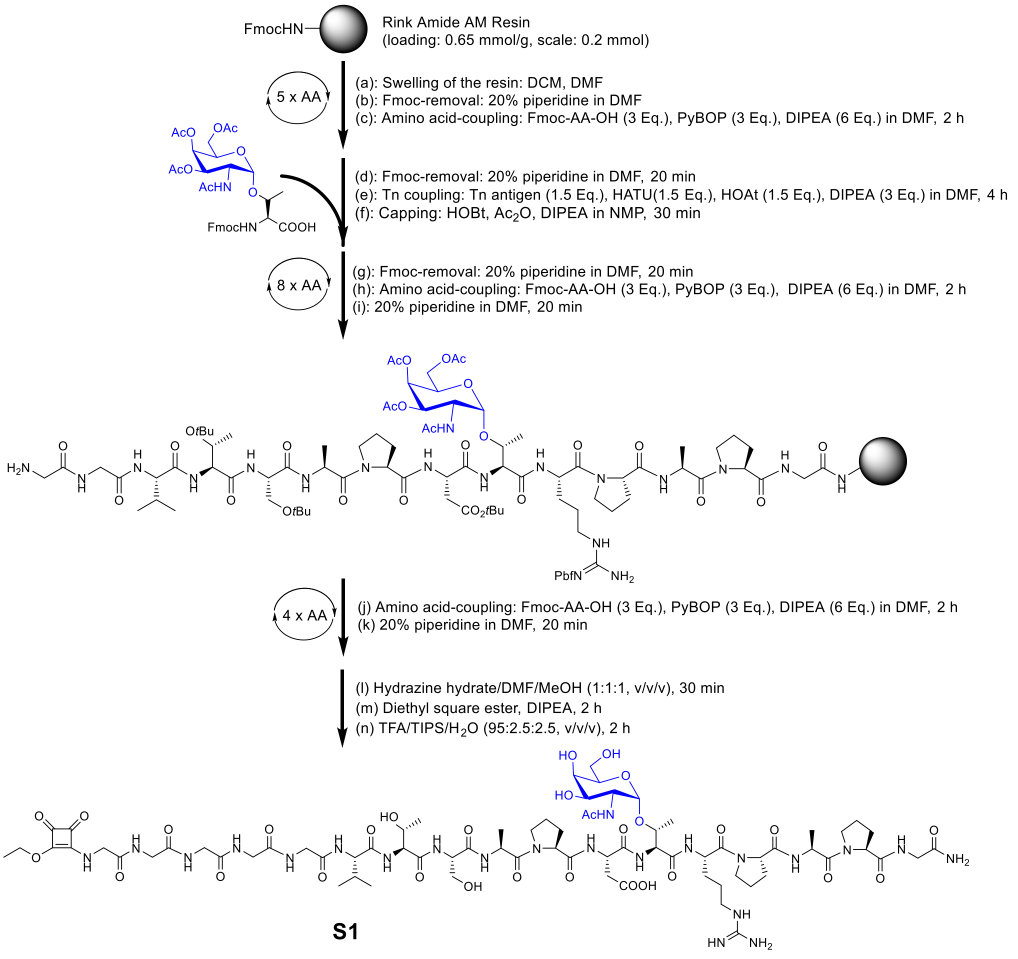

Scheme S1. The synthetic route of MUC1 glycopeptide squaric acid monoamide.

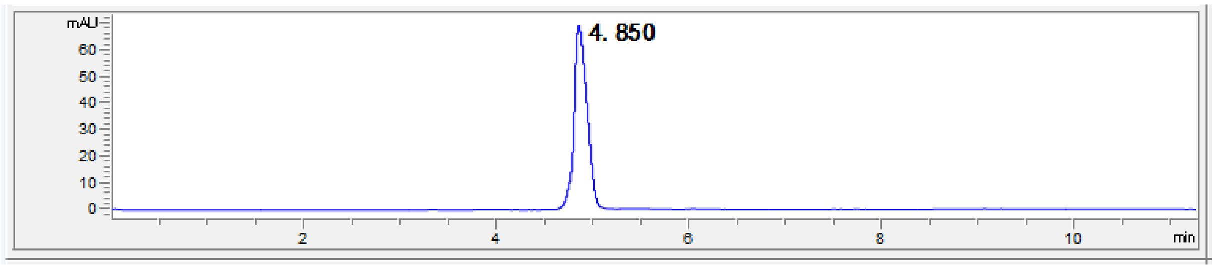

Figure S1. The HPLC chromatogram of MUC1 glycopeptide squaric acid monoamide. (column: Agilent C18), gradient: water/acetonitrile + 0.1% TFA, 0.0 min (95:5) → 15.0 min (10:90) → 20.0 min (0:100), Rt = 4.850 min. Related to Scheme S1.

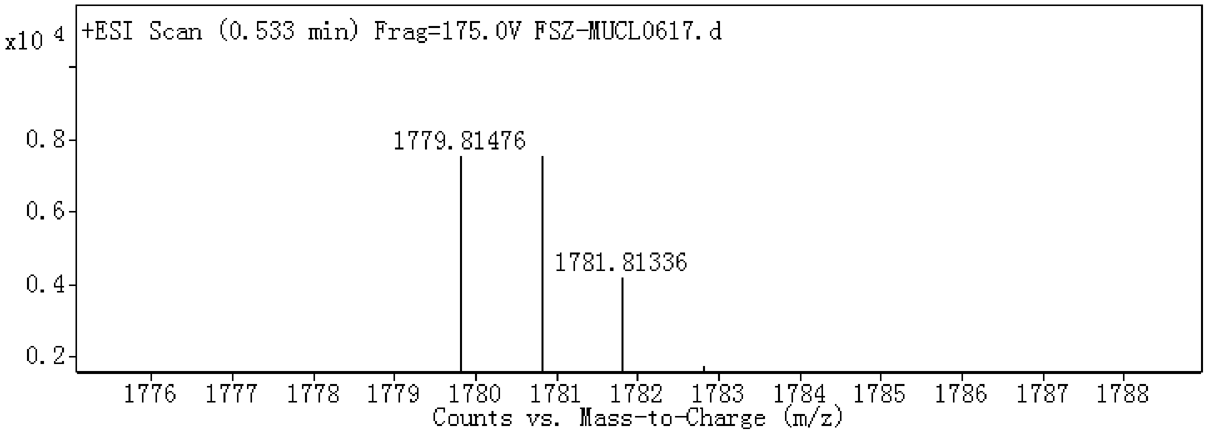

Figure S2. The HRMS data of MUC1 glycopeptide squaric acid monoamide. HRMS (ESI) calculated for $C_{73}H_{114}N_{22}O_{30}$ $[M+H]^+$: 1779.8114, found: 1779.8147. Related to Scheme S1.

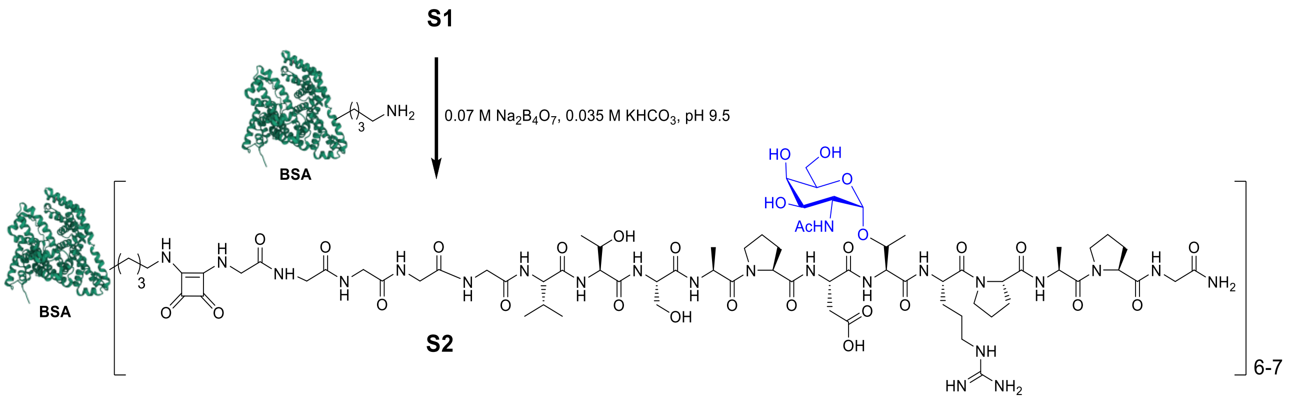

Scheme S2. Preparation of BSA-MUC1 conjugate.

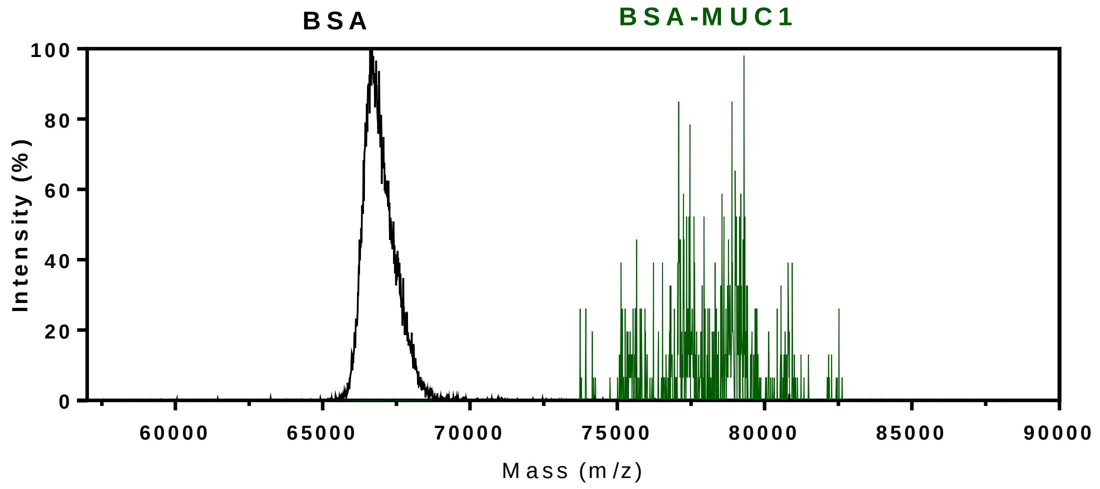

Figure S3. The MALDI-TOF-MS analysis of BSA-MUC1. The results showed that each BSA protein conjugated about 6-7 MUC1 glycopeptides. Related to Scheme S2.

1.3 Synthesis of the MUC1-TLR7a glycopeptide squaric acid monoamide

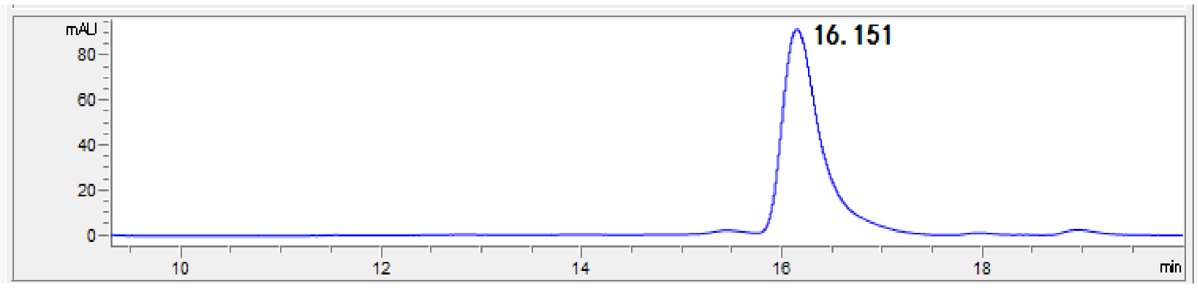

Figure S4. The HPLC chromatogram of MUC1-TLR7a glycopeptide squaric acid monoamide. (column: Agilent C18), gradient: water/acetonitrile + 0.1% TFA, 0.0 min (95:5) → 15.0 min (10:90) → 20.0 min (0:100), Rt = 16.151 min. Related to Scheme 1.

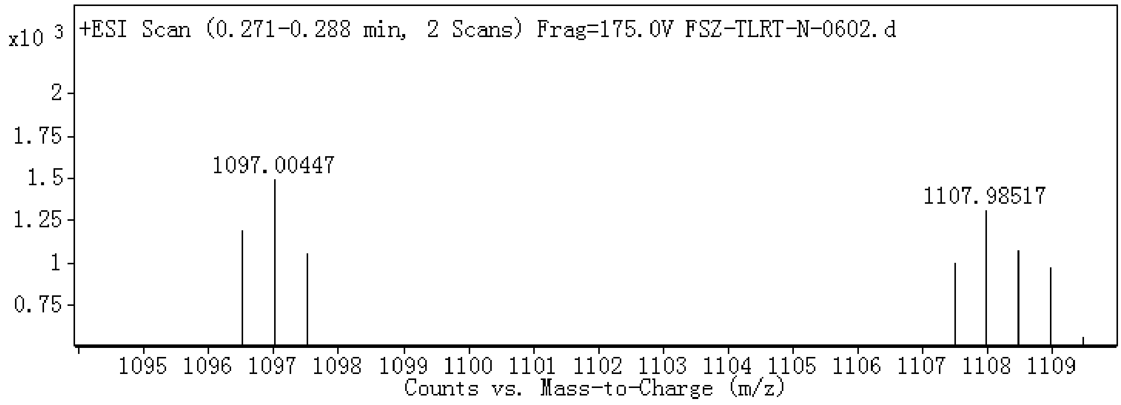

Figure S5. The HRMS data of MUC1-TLR7a glycopeptide squaric acid monoamide. calculated for $C_{93}H_{138}N_{28}O_{34}$ $[M+2H]^{+}$: 1097.0055, found: 1097.0044. Related to Scheme 1.

2. Evaluation of Vaccine Candidates

2.1 Vaccine formulation

Vaccines	Antigen	Adjuvant
**A**	BSA-MUC1 (118 μg)	/
**B**	BSA-MUC1 (118 μg)	TLR7a (3.59 μg)
**C**	BSA-MUC1 (118 μg)	Alum adjuvant (100 μL)
**D**	BSA-MUC1 (118 μg)	TLR7a (3.59 μg) and Alum adjuvant (100 μL)
**E**	BSA-MUC1-TLR7a (122 μg)	/
**F**	BSA-MUC1-TLR7a (122 μg)	Alum adjuvant (100 μL)

Table S1. The composition of each vaccine: the amount of each component in the table are used for one injection per mouse. All vaccine candidates contain 10 nmol MUC1 glycopeptide or 10 nmol TLR7 agonist.

2.2 Immunological evaluation

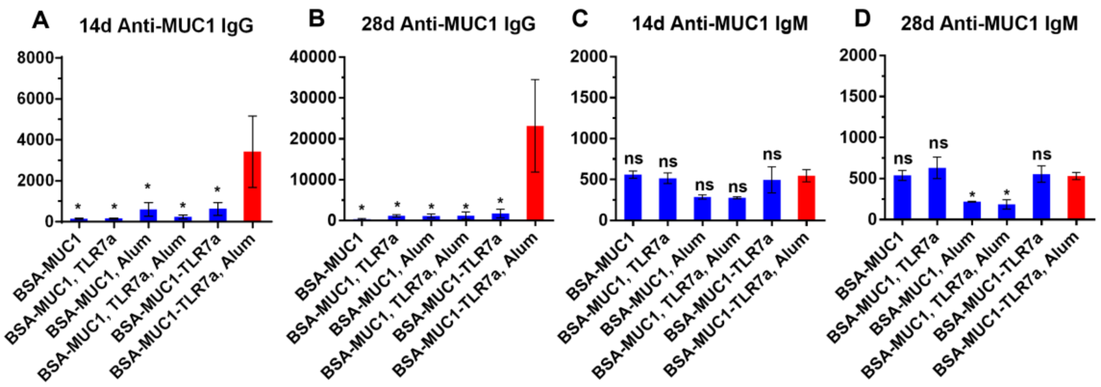

Figure S6. Anti-MUC1 IgG (A and B) and IgM (C and D) antibody titers were measured in serum samples from vaccinated mice collected on day 14 and 28. The data are expressed as the mean ± SEM. Asterisks show significant difference compared with BSA-MUC1-TLR7a/Alum group based on one-way ANOVA by Dunn's multiple comparison test (no significant difference, ns; $P \leq 0.05$, *). Related to Figure 2B.

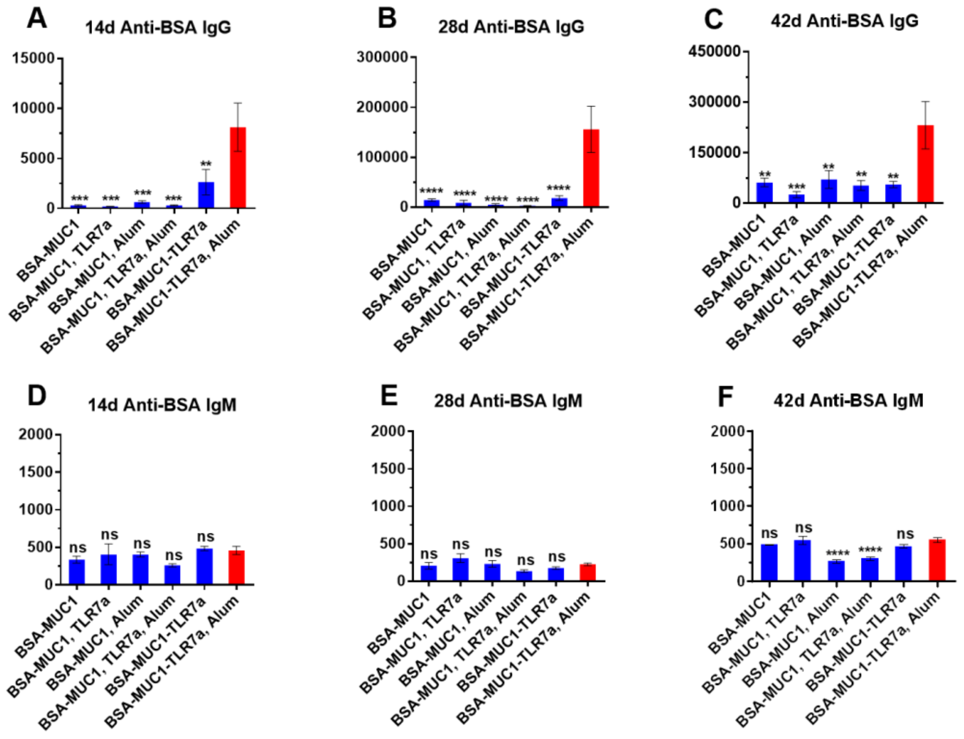

Figure S7. Anti-BSA IgG (A-C) and IgM (D-F) antibody titers were measured in serum samples from vaccinated mice collected on day 14, 28 and 42. The data are expressed as the mean ± SEM. Asterisks show significant difference compared with BSA-MUC1-TLR7a/Alum group by Dunn's multiple comparison test (no significant difference, ns; $P \leq 0.01$, **; $P \leq 0.001$, ***; $P \leq 0.0001$, ****). Related to Figure 2B.

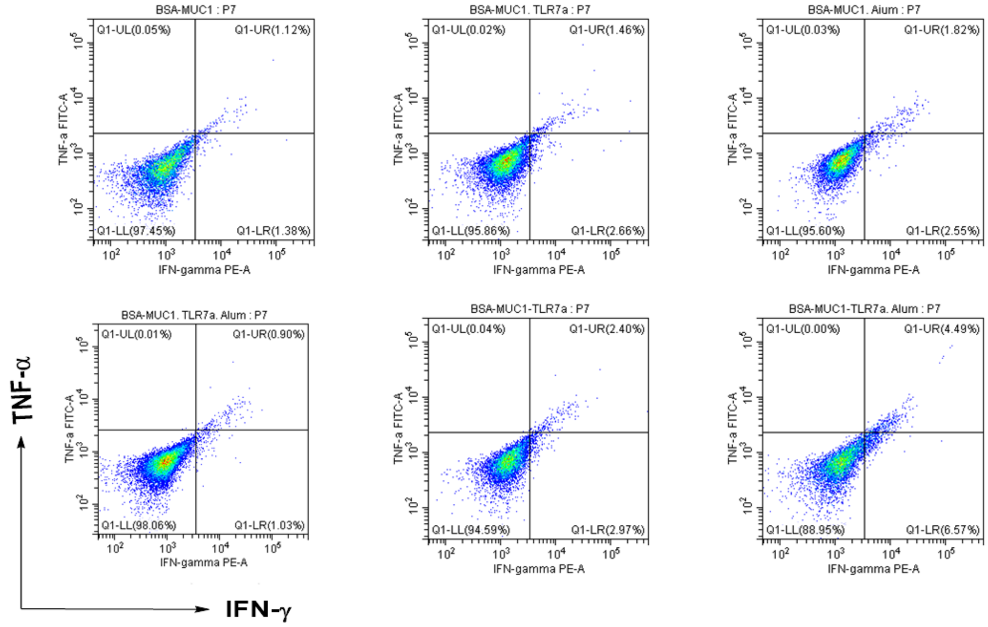

Figure S8. The lymphocytes in the spleen (day 42) were stimulated with the MUC1 glycopeptide for 18h, the results were analyzed by flow cytometry. Representative flow cytometry plots were shown in figure. Related to Figure 5A.

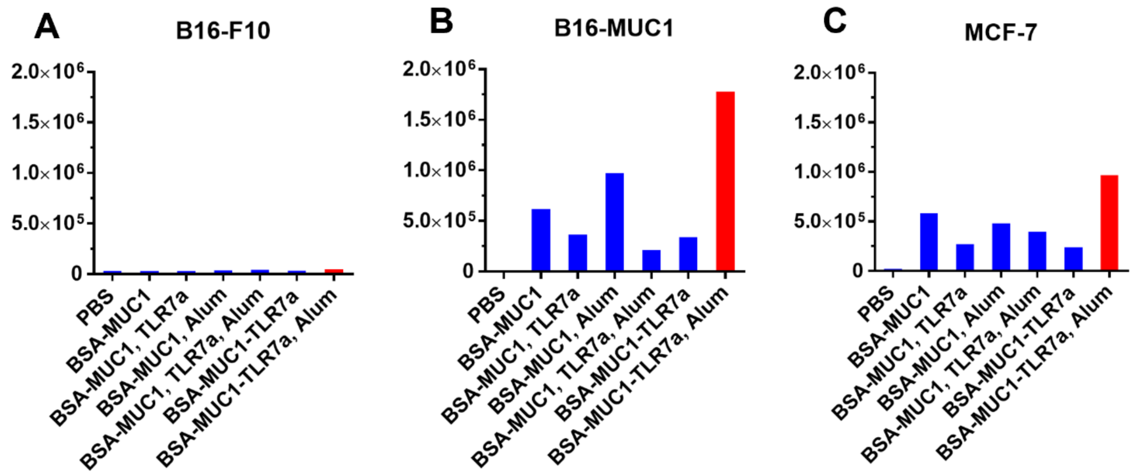

Figure S9. The binding of specific antibodies to (A) B16-F10, (B) B16-MUC1 and (C) MCF-7 cells (MFI, mean fluorescence intensity). Related to Figure 3.

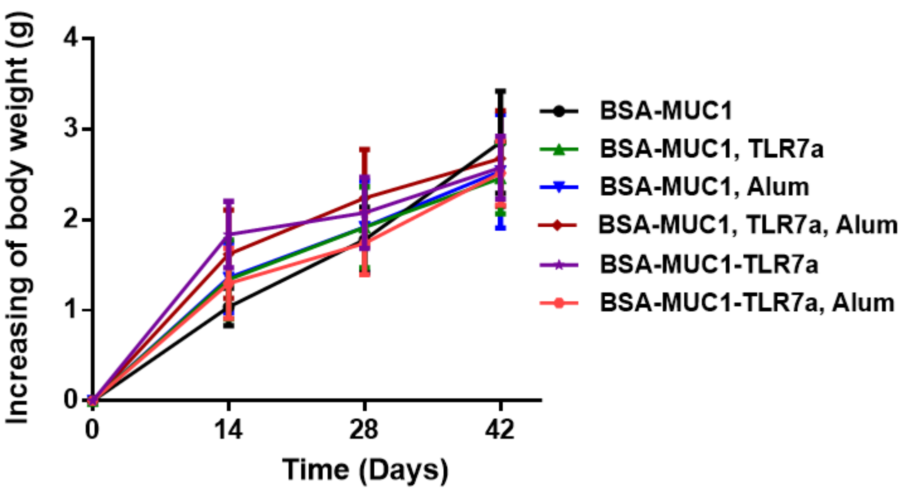

Figure S10. The increasing of body weight of mice. During the immunization period, no weight loss phenomena was observed in mice.

3 Biological testing methods

3.1 Immunization of mice and ELISA

Female SPF BALB/c mice aged 6–8 weeks were purchased from Huazhong Agricultural University, then mice were randomly divided into 6 groups with 5 mice per cage. The antigens of mice immunized were diluted in PBS (pH = 7.4). Mice were vaccinated by subcutaneous injection, then mice in each group were injected with the corresponding dosage form of the same dose once every 2 weeks. The sera were collected on days 0, 14, 28 and 42 after inoculation. Mice were euthanized on day 42 after vaccination, and the splenocytes were collected and subjected to ELISpot assay and intracellular cytokine staining. Mice used in the vaccination were conducted strictly in accordance with the principles of welfare and ethics of

medical laboratory animals. After collection, the blood samples were coagulated at room temperature for 1 h, and centrifuged at 4 °C and 6000 rpm for 8 minutes. The upper sera were stored at -80 °C (Chen et al., 2019).

For evaluation of antigen-specific antibodies, 1.25 μg Biotin-MUC1 glycopeptide and 11.6 μg avidin were diluted in 10 mL $NaHCO_3$ buffer (pH=9.5), then coated on 96-well plates at 100 μL per well overnight at 4 °C. The plates were washed with PBST (PBS with 0.05% Tween-20) 3 times, and then blocked with 1% casein in PBS at 200 μL per well for 1 h. Subsequently, the gradient diluted sera were added to 96-well plates at 37 °C for 1 h. Plates were washed 3 times and anti-mouse HRP-conjugated antibody diluted with PBS was added into wells for 1 h at 37 °C. Finally, plates were washed 4 times with PBST and color developed with TMB for 5 min at 25 °C, followed by 2 M H_2SO_4 stop solution. The absorptions at OD 450 nm was measured on BioTek SYNERGY H1 (Du et al., 2020).

For evaluation of cytokine levels, mice were vaccinated by intraperitoneal injection. The cytokine levels in sera were evaluated using ELISA kits (IL-6, BD Pharmingen) according to the manufacturer's protocol. Briefly, 96-well plates were coated with capture antibodies dissolved in the coating buffer per well and incubated overnight at 4 °C. The wells were then blocked with 10% FBS for 1 h at rt. After blocking, 100 μL/well of standard, sera, and control were added and incubated for 2 h at 28 °C. After washing, the working detector (detection antibody and SAV-HRP reagent) was added to each well. The plates were incubated for 1 h at 28 °C. Then, the plates were washed, and the tetramethyl benzidine (TMB) substrate solution was added. The reactions were stopped after 30 min at 28 °C with a stopping solution. The absorbance was measured at OD 450 nm n BioTek SYNERGY H1.

3.2 Cell culture and FACS Assay

B16-MUC1 cells and B16-F10 were cultured in RPMI-1640 containing 10% FBS and 1% antibiotic, and MCF-7 cells were cultured in DMEM containing 10% FBS and 1% antibiotic. For the FACS assay, cells were harvested and washed with FACS buffer (PBS containing 10% FBS and 0.1% sodium azide). The cells were incubated with pooled sera of each group (1:50 dilution in FACS buffer) for 1 h at 4 °C. Then cells were washed three times with FACS buffer and incubated with FITC-linked goat antimouse kappa antibody (Alexa Fluor 488-conjugated Goat Anti-Mouse IgG (H+L), Jackson ImmunoResearch, diluted 1:50) for 30 min at 4 °C. After washing three times with FACS buffer, the cells were added to the PBS and analyzed by flow cytometry (Du et al., 2019).

3.3 Complement dependent cytotoxicity (CDC) assay

MCF-7 cells were seeded in the wells of the 96-well plate (8000 cells per well). After incubation at 37 °C overnight, the plate was washed with the medium, then incubated with sera (diluted 1:30 in medium, 100 μL per well) from vaccinated mice for 1 h. After washing with PBS solution, the rabbit complement (diluted 1:50 in medium, 100 μL per well) in 1% BSA/PBS was added. The rabbit complement inactivated by treatment at 65 °C for 30 min was used as the control. After incubation for 2 h, 0.5% MTT solution in PBS was added (50 μL/well) and incubated for 2 h. After removing the medium, DMSO was added (150 μL/well) and the absorption was analyzed at the wavelength of 490 nm (Yin et al., 2021).

Cell viability (%) = (Experimental/Control) × 100

3.4 Cytotoxic T lymphocyte assay (CTL) assay

The freshly isolated splenocytes on day 42 (1×10^6 cells/well) were added and co-incubated with the MCF-7 cells (1×10^6 cells/well) in RPMI-1640 for 12 h. Finally, the effector cell-mediated cytotoxicity to target cells was examined by LDH assay according to the manufacture's protocol (Beyotime Biotechnology). Each plate was centrifuged at 250 g for 4 min, then 120 μL of the cell-free supernatant was transferred to the wells of another 96-well enzymatic assay plate containing LDH assay reagents (60 μL/well). The 96-well plates were incubated at rt protected from light for 30 min. The absorptions of these plates were read at 490 nm wavelength using a microplate reader. In the meantime, the spontaneous LDH release values were determined by incubating tumor cells alone or splenocytes alone, respectively. The maximum LDH release values were determined by incubating tumor cells in RPMI-1640 containing lysis solution without FBS (Du et al., 2020).

3.5 Enzyme-linked immunospot (ELISpot) assay

The antigen specific T cell responses were detected by ELISpot assays. The splenocytes were isolated 14 days after receiving final vaccination, then the assays were performed using Mouse IFN-γ precoated ELISpot kit (Dakewe, 2210005). In brief, the splenocytes were seeded at 1×10^6 cells per well and stimulated with MUC1 glycopeptide (1 μg per well) *in vitro*. After 18 h, biotinylated antibody and streptavidin-HRP were added into plates, and then the spots were developed by AEC solution at 37 ºC for 15 min, finally the spots were counted using ELISpot reader.

3.6 Intracellular cytokine staining (ICS)

Similar to ELISpot assay, mouse splenocytes were stimulated with MUC1 glycopeptide, the cells were incubated with brefeldin A and monensin overnight after four hours of stimulation. After washing, cells were stained with APC/Cyanine7 anti-mouse CD3 and APC anti-mouse CD8a (BioLegend) for 30 min, then cells were fixed and permeabilized with PE anti-mouse IFN-γ and FITC anti-mouse TNF-α (BioLegend). Cells were analyzed and gated on a CytoFLEX S flow cytometer (Beckman Coulter).

3.7 Evaluation of anti-tumor immune response in mice

B16-MUC1 tumor cells (5×10^5) were administered subcutaneously into the right flank of C57BL/6 female mice aged 4-6 weeks. Twelve days after transplant, the diameter of the tumor reached 5 mm. (five mice per group). Mice were given peritumoral injections of vaccine candidates every four days and a total of three injections were conducted. Tumor sizes were detected every two days (Li et al., 2019). Volume was calculated by $0.5 \times \text{length} \times \text{width}^2$.

3.8 Statistical analysis and references

Data in the figures were analyzed using GraphPad Prism 6. Except as specifically stated, all values were mean and error bars were SEM. Significance of groups in comparison was analyzed using one-way ANOVA or two-way ANOVA by Dunn's multiple comparison test. Asterisks showed significant difference (no significant difference, ns; $P \leq 0.05$, *; $P \leq 0.01$, **; $P \leq 0.001$, ***; $P \leq 0.0001$, ****).

References

Chen, X. Z., Zhang, R. Y., Wang, X. F., Yin, X. G., Wang, J., Wang, Y. C., et al. (2019). Peptide-free Synthetic Nicotine Vaccine Candidates with α-Galactosylceramide as Adjuvant. *Mol Pharm* 16, 1467–1476. doi: 10.1021/acs.molpharmaceut.8b01095.

Du, J. J., Zou, S. Y., Chen, X. Z., Xu, W. B., Wang, C. W., Zhang, L., et al. (2019). Liposomal Antitumor Vaccines Targeting Mucin 1 Elicit a Lipid-Dependent Immunodominant Response. *Chem - An Asian J* 14, 2116–2121. doi: 10.1002/asia.201900448.

Du, J. J., Wang, C. W., Xu, W. B., Zhang, L., Tang, Y. K., Zhou, S. H., et al. (2020). Multifunctional Protein Conjugates with Built-in Adjuvant (Adjuvant-Protein-Antigen) as Cancer Vaccines Boost Potent Immune Responses. *iScience* 23, 100935. doi: 10.1016/j.isci.2020.100935.

Li, M., Wang, Z., Yan, B., Yin, X., Zhao, Y., Yu, F., et al. (2019). Design of a MUC1-based tricomponent vaccine adjuvanted with FSL-1 for cancer immunotherapy. *Medchemcomm* 10, 2073–2077. doi: 10.1039/c9md00254e.

Yin, X.-G., Lu, J., Wang, J., Zhang, R.-Y., Wang, X.-F., Liao, C.-M., et al. (2021). Synthesis and Evaluation of Liposomal Anti-GM3 Cancer Vaccine Candidates Covalently and Noncovalently Adjuvanted by αGalCer. *J Med Chem* 64, 1951–1965. doi: 10.1021/acs.jmedchem.0c01186.
